# Commentary on “A Population‐Based Correlation Analysis Between Hemoglobin A1c and Hemoglobin Levels”

**DOI:** 10.1111/1753-0407.70087

**Published:** 2025-04-28

**Authors:** Youyuan Hu, Tinghua Zhang

**Affiliations:** ^1^ Department of Pathology The Second People's Hospital of Huaihua Huaihua Hunan China; ^2^ Department of Clinical Laboratory The Second People's Hospital of Huaihua Huaihua Hunan China

**Keywords:** estrogen, hemoglobin, hemoglobin A1c

To the Editor,

We read with interest the study by Zhang et al. [1], which explored the gender‐ and age‐specific associations between hemoglobin A1c (HbA1c) and hemoglobin levels in a large Chinese cohort. While the authors provide valuable insights into the potential role of estrogen in modulating HbA1c, several methodological and interpretative limitations warrant discussion to strengthen the validity and generalizability of their conclusions.

The study's reliance on health examination data from Southwest China raises concerns about external validity. Regional variations in genetic, dietary, and socioeconomic factors may influence hemoglobin and HbA1c dynamics, limiting extrapolation to global populations. Furthermore, while the authors adjusted for basic covariates (e.g., age, gender), critical confounders such as iron status, inflammation markers (e.g., C‐reactive protein), and nutritional deficiencies—known to affect both hemoglobin and HbA1c—were omitted. For instance, iron deficiency anemia disproportionately impacts women and could confound the observed correlations [2].

The use of a binary age cutoff (≤ 45 vs. > 45 years) to approximate menopausal status is problematic. Menopause timing varies widely across individuals and ethnicities, with a significant proportion of women experiencing it after 45. Without direct assessment of hormonal levels (e.g., estradiol, FSH) or menstrual history, the assumption that age alone accurately reflects estrogen status risks misclassification bias. This may obscure nuanced relationships, particularly in perimenopausal populations.

Although generalized additive models (GAMs) effectively capture non‐linear trends, the absence of model diagnostics (e.g., residual plots, goodness‐of‐fit metrics) undermines confidence in their robustness. Additionally, the reported Pearson's correlation coefficients (figure 1) appear incongruent with the non‐linear splines, suggesting potential overfitting. A sensitivity analysis comparing GAMs with simpler linear models adjusted for spline terms would clarify whether the observed associations are artifacts of modeling complexity.

The hypothesis linking estrogen decline to elevated HbA1c in postmenopausal women, while plausible, remains speculative. The study lacks direct measurements of estrogen or markers of insulin resistance (e.g., HOMA‐IR), relying instead on indirect epidemiological inferences. Longitudinal data or subgroup analyses stratified by hormone replacement therapy (HRT) use could strengthen causal inference. Notably, HRT's glucose‐lowering effects in diabetic women—a finding that aligns with the authors' hypothesis but was not leveraged in this cross‐sectional design [3].

The gender‐specific reference intervals (RIs) for hemoglobin, though aligned with WHO criteria, may not account for altitude‐related variations in hemoglobin, prevalent in Southwest China's highland populations. This oversight could skew RIs, particularly in men residing at higher elevations.

## Recommendations

Future studies should incorporate direct hormonal assessments, expand geographic diversity, and adjust for hematological confounders (e.g., ferritin, transferrin saturation). Prospective designs tracking HbA1c and hemoglobin pre‐ and post‐menopause would better elucidate temporal relationships. Lastly, validating RIs against altitude‐adjusted norms would enhance clinical utility.

While Zhang et al. provide a foundational understanding of HbA1c‐hemoglobin interactions, addressing these limitations could refine risk stratification and therapeutic strategies in diverse populations.

## Conflicts of Interest

The authors declare no conflicts of interest.
